# Outcomes of relapsed clinical stage I versus de novo metastatic testicular cancer patients: an analysis of the IGCCCG Update database

**DOI:** 10.1038/s41416-023-02443-3

**Published:** 2023-09-30

**Authors:** Jakob Lauritsen, Nicolas Sauvé, Alexey Tryakin, Di Maria Jiang, Robert Huddart, Daniel Y. C. Heng, Angelika Terbuch, Eric Winquist, Michal Chovanec, Marcus Hentrich, Christian D. Fankhauser, Jonathan Shamash, Xavier Garcia del Muro, David Vaughn, Axel Heidenreich, Cora N. Sternberg, Christopher Sweeney, Andrea Necchi, Carsten Bokemeyer, Mikkel Bandak, Abolghassem Jandari, Laurence Collette, Silke Gillessen, Joerg Beyer, Gedske Daugaard

**Affiliations:** 1 Department of Oncology, Copenhagen University Hospital, Rigshospitalet, Denmark; 2 European Organisation for Research and Treatment of Cancer (EORTC), Brussels, Belgium; 3 Department of Chemotherapy, N.N.Blokhin Russian Cancer Research Center, Moscow, Russia; 4 Division of Medical Oncology and Hematology, Princess Margaret Cancer Centre, University of Toronto, Toronto, ON Canada; 5 Institute of Cancer Research and Royal Marsden Hospital, Downs Road, Sutton, Surrey UK; 6 Division of Medical Oncology, Tom Baker Cancer Centre, University of Calgary, Calgary, Alberta Canada; 7 Division of Oncology, Medical University of Graz, Graz, Austria; 8 Department of Oncology, University of Western Ontario and London Health Sciences Centre, London, ON Canada; 9 2nd Department of Oncology, Faculty of Medicine, Comenius University, National Cancer Institute, Bratislava, Slovakia; 10 Department of Hematology/Oncology, Red Cross Hospital, Munich, Germany; 11 University of Zurich, Zurich, Switzerland; 12 University of Lucerne, Lucerne, Switzerland; 13 Department of Oncology, St Bartholomew’s Hospital, London, UK; 14 Department of Medical Oncology, Catalan Institute of Oncology, Barcelona, Spain; 15 Division of Hematology/Oncology, Abramson Cancer Center at the University of Pennsylvania, Philadelphia, PA USA; 16 Department of Urology, Uro-Oncology, Robot Assisted and Specialized Urologic Surgery, University Hospital Cologne, Cologne, Germany; 17 Englander Institute for Precision Medicine, Sandra and Edward Meyer Cancer Center, Weill Cornell Medicine, New York-Presbyterian, New York, NY USA; 18 Dana-Farber Cancer Institute, Harvard Medical School, Boston, USA; 19 Vita-Salute San Raffaele University, Department of Medical Oncology, IRCCS San Raffaele Hospital, Milan, Italy; 20 Department of Oncology, Hematology and BMT with section Pneumonology, University Hospital Hamburg-Eppendorf, Hamburg, Germany; 21 Department of Consulting and Research, International Drug Development Institute, Louvain-la-Neuve, Belgium; 22 Oncology Institute of Southern Switzerland, EOC, Bellinzona, Switzerland; 23 University of Southern Switzerland, Lugano, Switzerland; 24 Department of Medical Onccology, Inselspital, Bern University Hospital, University of Bern, Bern, Switzerland

**Keywords:** Medical research, Germ cell tumours

## Abstract

**Background:**

Active surveillance after orchiectomy is the preferred management in clinical stage I (CSI) germ-cell tumours (GCT) associated with a 15 to 30% relapse rate.

**Patients and methods:**

In the IGCCCG Update database, we compared the outcomes of gonadal disseminated GCT relapsing from initial CSI to outcomes of patients with de novo metastatic GCT.

**Results:**

A total of 1014 seminoma (Sem) [298 (29.4%) relapsed from CSI, 716 (70.6%) de novo] and 3103 non-seminoma (NSem) [626 (20.2%) relapsed from CSI, 2477 (79.8%) de novo] were identified. Among Sem, no statistically significant differences in PFS and OS were found between patients relapsing from CSI and de novo metastatic disease [5-year progression-free survival (5y-PFS) 87.6% versus 88.5%; 5-year overall survival (5y-OS) 93.2% versus 96.1%). Among NSem, PFS and OS were higher overall in relapsing CSI patients (5y-PFS 84.6% versus 80.0%; 5y-OS 93.3% versus 88.7%), but there were no differences within the same IGCCCG prognostic groups (HR = 0.89; 95% CI: 0.70–1.12). Relapses in the intermediate or poor prognostic groups occurred in 11/298 (4%) Sem and 112/626 (18%) NSem.

**Conclusion:**

Relapsing CSI GCT patients expect similar survival compared to de novo metastatic patients of the same ICCCCG prognostic group. Intermediate and poor prognosis relapses from initial CSI expose patients to unnecessary toxicity from more intensive treatments.

## Introduction

Different treatment options may be offered to patients with germ cell tumours (GCT) localised to one or both testicles [clinical stage I disease (CSI)], depending on prognostic factors and institutional preferences [1–3]. In contrast to adjuvant carboplatin in seminoma (Sem) or combination chemotherapy or retroperitoneal lymph node dissection in non-seminoma (NSem), active surveillance without adjuvant treatment has become the preferred choice at many centres due to the low risk of relapse and high survival rates even if relapse occurs [5–7].

Around 70% of GCT patients present with CSI disease, of whom 15–30% will experience a relapse if followed on an active surveillance programme [4, 5]. The overall relapse rate depends on prognostic factors and whether active surveillance is chosen for all patients or only for low-risk patients, with high-risk patients being offered adjuvant treatment, as seen in many institutions [8]. Traditionally, patients with relapse from CSI are treated as patients with de novo metastatic disease and classified into prognostic groups as per IGCCCG [8–10].

Ideally, relapsing CSI patients should be detected early and with a low tumour burden, thus harbouring a better prognosis overall compared to patients with de novo metastatic disease who often present with more advanced disease in the intermediate and/or poor prognosis IGCCCG category [4, 5, 7, 9]. While this is true for published series of CSI patients on prospective and well-structured active surveillance programmes, in whom the survival is close to 100%, it is less clear if these same results can be replicated, where follow-up might be less stringent. To study the presentation and distribution across IGCCCG prognostic groups as well as the survival probabilities in relapsing CSI patients as compared to de novo metastatic patients in a more real-world scenario, we used the database of the IGCCCG Update consortium warehouse, consisting of large multicenter and multinational cohorts [11, 12].

## Methods

We identified eligible patients for the present analysis in the IGCCCG-update data for whom information on the disease stage at initial presentation as well as information at the time of metastatic disease was available. The collection of the IGCCCG-update data warehouse has previously been presented and is an aggregate of prospectively or retrospectively collected data (cohort, registry or trials) from 30 institutions or collaborative groups in Europe, North America, and Australia [11, 12]. The current analyses were not pre-planned prior to the creation of the data warehouse.

**Fig. 1:**
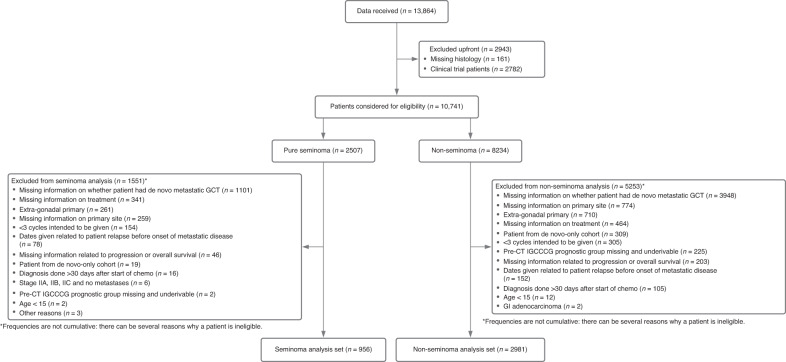
Flowchart of included patients.

A flowchart of patients included in this study is presented in Fig. 1. To ensure comparable cohorts, patients with extragonadal disease were excluded as these patients could only have de novo metastatic disease. To reduce the inherent selection bias in this analysis, patient cohorts that exclusively provided de novo metastatic patients were also excluded, as we suspected that these were primarily from tertiary referral clinics, which did not manage patients with CSI disease. As one of the objectives was to describe the treatments of GCT patients relapsing from initial CSI given in routine clinical practice, patients treated in the context of a clinical trial were excluded as well.

### Aims of the study and endpoints

The first aim of the study was to assess the stage of relapsing CSI, as we suspected that outside structured follow-up programmes, more CSI patients might relapse with more advanced tumour stages than had been reported previously. The second aim was to describe the treatment of relapsing CSI patients and their treatment outcomes compared to de novo metastatic patients.

Stage at relapse was classified according to IGCCCG prognostic groups [10]. Progression-free survival (PFS) was defined from the start of chemotherapy to progression, defined by radiological progression, unequivocal tumour marker increase, or death, whichever came first. Overall survival (OS) was defined as the time from the start of chemotherapy to death of any cause. We used the PFS and OS at 5 years as the primary endpoints to harmonise the duration of follow-up across data sources since most events had occurred by this time.

### Statistical methods

Descriptive summary statistics of baseline characteristics are reported by histology, separately for CSI-relapsing and de novo metastatic patients: median, range and first and third quartiles for continuous variables, and frequencies for categorical variables. The number of patients from cohorts that did not provide the variable is given separately. Survival estimates are reported via the Kaplan–Meier method. 95% confidence intervals (95% CI) are provided via log-log transform and the Greenwood variance estimate [13]. A Cox proportional hazard model stratified on IGCCCG prognostic groups is fitted, adjusting for de novo status (yes/no). The hazard ratio (HR) for de novo status is given with the corresponding 95% CI.

## Results

Out of 6920 patients assessed for eligibility, 1014 with Sem and 3103 with NSem were eligible for this not-pre-planned analysis. Reasons for ineligibility are listed in Fig. 1. The most common ineligibility criterion was extragonadal primary (*N* = 845, 69.1% of all excluded patients).

Patients with NSem relapsing from CSI were less likely to present with intermediate or poor risk metastatic disease as compared to de novo metastatic patients. Compared to de novo metastatic patients, more patients with Nsem relapsing from CSI presented within the good prognosis IGCCCG group (82.1% versus 51.4%, Table 1), had lower median values for AFP (26.9 ng/mL versus 64.4 ng/mL), lower median values for HCG (18.5 U/l versus 68 U/l), less frequent lung metastases (24.3% versus 36.3%) and less frequent non-pulmonary organ metastases (4.8% versus 11.4%) (Table 2). Notably, the remaining 11.0% and 6.9% of NSem patients with CSI relapsed with intermediate or poor prognostic features, respectively.

**Table 1:** Baseline characteristics.

	Non-seminoma			Seminoma		
	Relapsed stage 1 (*N* = 626)	De novo (*N* = 2477)	Total (*N* = 3103)	Relapsed stage 1 (*N* = 298)	De novo (*N* = 716)	Total (*N* = 1014)
**Age at diagnosis of metastatic disease (years)**						
**Median**	30.0	29.0	29.0	37.1	38.0	38.0
**Range**	15.0–70.0	15.0–76.0	15.0–76.0	17.1–74.7	16.0–77.6	16.0–77.6
**Q1-Q3**	25.1–36.0	24.0–36.0	24.0–36.0	31.6–43.4	32.0–45.2	32.0–45.0
**Missing**	0	12	12	1	3	4
**Original IGCCCG prognostic groups**						
**Good**	514 (82.1)	1274 (51.4)	1788 (57.6)	287 (96.3)	690 (96.4)	977 (96.4)
**Intermediate**	69 (11.0)	693 (28.0)	762 (24.6)	11 (3.7)	26 (3.6)	37 (3.6)
**Poor**	43 (6.9)	510 (20.6)	553 (17.8)			
**Pre-chemo AFP levels (ng/mL)**						
**Median**	26.9	64.4	50.8	3.3	3.0	3.1
**Range**	0.0–2420000.0	0.0–2007390.0	0.0–2420000.0	0.0–9.7	0.0–16.9	0.0–16.9
**Q1-Q3**	5.0–100.4	7.3–550.6	6.9–409.0	2.1–5.0	2.0–4.8	2.0–4.8
**Missing**	172 (27.5)	434 (17.6)	606 (19.6)	137 (46.0)	231 (32.3)	368 (36.3)
**Pre-chemo HCG levels (U/L)**						
**Median**	18.5	68.0	49.0	3.0	2.1	2.6
**Range**	0.0–1505610.0	0.0–35000000.0	0.0–35000000.0	0.0–36700.0	0.0–276043.0	0.0–276043.0
**Q1-Q3**	2.0–114.0	4.2–1556.0	4.0–887.0	1.0–19.0	1.0–15.8	1.0–16.6
**Missing**	190 (30.1)	436 (17.6)	626 (20.2)	107 (36.0)	190 (26.6)	297 (29.3)
**Pre-chemo LDH/ULN levels**						
**Median**	0.9	1.2	1.1	1.2	1.2	1.2
**Range**	0.3–27.4	0.0–71.0	0.0–71.0	0.5–52.1	0.0–101.3	0.0–101.3
**Q1-Q3**	0.7–1.4	0.8–2.4	0.8–2.2	0.8–2.1	0.8–2.6	0.8–2.4
**Missing**	335 (53.6)	508 (20.6)	843 (27.2)	133 (44.7)	201 (28.1)	334 (33.0)
**Pre-chemo AFP levels (categorised)**						
**<1000 ng/mL**	419 (68.2)	1626 (66.7)	2045 (67.0)	161 (55.3)	485 (68.5)	646 (64.7)
**1000–10,000 ng/mL**	24 (3.9)	292 (12.0)	316 (10.4)	0 (0.0)	0 (0.0)	0 (0.0)
**>10,000 ng/mL**	11 (1.8)	125 (5.1)	136 (4.5)	0 (0.0)	0 (0.0)	0 (0.0)
**Missing**	172 (27.5)	434 (17.6)	606 (19.6)	137 (46.0)	231 (32.3)	368 (36.3)
**Pre-chemo HCG levels (categorised)**						
**<5000 IU/L**	413 (67.3)	1667 (68.4)	2080 (68.2)	188 (64.6)	520 (73.4)	708 (70.9)
**5000–50,000 IU/L**	13 (2.1)	179 (7.3)	192 (6.3)	3 (1.0)	5 (0.7)	8 (0.8)
**>50,000 IU/L**	10 (1.6)	195 (8.0)	205 (6.7)	0 (0.0)	1 (0.1)	1 (0.1)
**Missing**	190 (30.1)	436 (17.6)	626 (20.2)	107 (36.0)	190 (26.6)	297 (29.3)
**Pre-chemo LDH levels (categorised)**						
≤**1.5 UNL**	225 (38.5)	1165 (48.8)	1390 (46.8)	97 (34.4)	299 (42.5)	396 (40.2)
**1.5–10 UNL**	64 (11.0)	736 (30.8)	800 (26.9)	65 (23.0)	194 (27.6)	259 (26.3)
**>10 UNL**	2 (0.3)	68 (2.8)	70 (2.4)	3 (1.1)	22 (3.1)	25 (2.5)
**Missing**	335 (53.6)	508 (20.6)	843 (27.2)	133 (44.7)	201 (28.1)	334 (33.0)
**Progression/relapse**						
**No progression**	516 (82.4)	1918 (77.4)	2434 (78.4)	259 (86.9)	616 (86.0)	875 (86.3)
**Progression in the first 3 years**	82 (13.1)	434 (17.5)	516 (16.6)	33 (11.1)	75 (10.5)	108 (10.7)
**Progression after 3 years**	28 (4.5)	125 (5.0)	153 (4.9)	6 (2.0)	25 (3.5)	31 (3.1)
**Overall survival**						
**Alive**	573 (91.5)	2154 (87.0)	2727 (87.9)	276 (92.6)	674 (94.1)	950 (93.7)
**Death in the first 3 years**	36 (5.8)	232 (9.4)	268 (8.6)	13 (4.4)	21 (2.9)	34 (3.4)
**Death after 3 years**	17 (2.7)	91 (3.7)	108 (3.5)	9 (3.0)	21 (2.9)	30 (3.0)
**Treatment period**						
**<1995**	89 (14.2)	272 (11.0)	361 (11.6)	37 (12.4)	65 (9.1)	102 (10.1)
**1995–1999**	120 (19.2)	406 (16.4)	526 (17.0)	51 (17.1)	134 (18.7)	185 (18.2)
**2000–2004**	166 (26.5)	617 (24.9)	783 (25.2)	73 (24.5)	199 (27.8)	272 (26.8)
**2005–2009**	152 (24.3)	697 (28.1)	849 (27.4)	80 (26.8)	198 (27.7)	278 (27.4)
**2010–2013**	99 (15.8)	485 (19.6)	584 (18.8)	57 (19.1)	120 (16.8)	177 (17.5)

*AFP* alpha-fetoprotein, *HCG* human chorionic gonadotropin, *IGCCCG* International Germ Cell Cancer Collaborative Group, *LDH* lactate dehydrogenase, *ULN* upper limit of normal.

**Table 2:** Metastatic sites.

	Non-seminoma			Seminoma		
	Relapsed stage 1 (*N* = 626)	De novo (*N* = 2477)	Total (*N* = 3103)	Relapsed stage 1 (*N* = 298)	De novo (*N* = 716)	Total (*N* = 1014)
**Presence of non-pulmonary visceral metastases (NPVM)**						
**Known absent**	590 (94.2)	2178 (87.9)	2768 (89.2)	287 (96.3)	687 (95.9)	974 (96.1)
**Known present**	30 (4.8)	283 (11.4)	313 (10.1)	11 (3.7)	24 (3.4)	35 (3.5)
**Assumed present (for intermediate seminoma with missing NPVM information)**	0 (0.0)	0 (0.0)	0 (0.0)	0 (0.0)	2 (0.3)	2 (0.2)
**Assumed absent (for good seminoma and good/intermediate non-seminoma with missing NPVM information)**	6 (1.0)	16 (0.6)	22 (0.7)	0 (0.0)	3 (0.4)	3 (0.3)
**Metastases in the bone**						
**Known absent**	613 (97.9)	2409 (97.3)	3022 (97.4)	292 (98.0)	700 (97.8)	992 (97.8)
**Known present**	7 (1.1)	51 (2.1)	58 (1.9)	6 (2.0)	11 (1.5)	17 (1.7)
**Assumed absent (for good seminoma and good/intermediate non-seminoma with missing NPVM information)**	0 (0.0)	0 (0.0)	0 (0.0)	0 (0.0)	0 (0.0)	0 (0.0)
**Missing**	6 (1.0)	17 (0.7)	23 (0.7)	0 (0.0)	5 (0.7)	5 (0.5)
**Metastases in the brain**						
**Known absent**	616 (98.4)	2410 (97.3)	3026 (97.5)	297 (99.7)	711 (99.3)	1008 (99.4)
**Known present**	4 (0.6)	50 (2.0)	54 (1.7)	1 (0.3)	0 (0.0)	1 (0.1)
**Assumed absent (for good seminoma and good/intermediate non-seminoma with missing NPVM information)**	0 (0.0)	0 (0.0)	0 (0.0)	0 (0.0)	0 (0.0)	0 (0.0)
**Missing**	6 (1.0)	17 (0.7)	23 (0.7)	0 (0.0)	5 (0.7)	5 (0.5)
**Metastases in the liver**						
**Known absent**	602 (96.2)	2255 (91.0)	2857 (92.1)	296 (99.3)	706 (98.6)	1002 (98.8)
**Known present**	18 (2.9)	206 (8.3)	224 (7.2)	2 (0.7)	6 (0.8)	8 (0.8)
**Assumed absent (for good seminoma and good/intermediate non-seminoma with missing NPVM information)**	0 (0.0)	0 (0.0)	0 (0.0)	0 (0.0)	0 (0.0)	0 (0.0)
**Missing**	6 (1.0)	16 (0.6)	22 (0.7)	0 (0.0)	4 (0.6)	4 (0.4)
**Metastases in abdominal lymph nodes**						
**No**	94 (26.7)	197 (12.1)	291 (14.7)	29 (15.6)	20 (4.1)	49 (7.3)
**Yes**	252 (71.6)	1421 (87.0)	1673 (84.2)	157 (84.4)	463 (95.3)	620 (92.3)
**Missing**	280 (44.7)	859 (34.7)	1139 (36.7)	112 (37.6)	233 (32.5)	345 (34.0)
**Presence of lung metastases**						
**No**	468 (74.8)	1563 (63.1)	2031 (65.5)	276 (92.6)	672 (93.9)	948 (93.5)
**Yes**	152 (24.3)	899 (36.3)	1051 (33.9)	22 (7.4)	39 (5.4)	61 (6.0)
**Missing**	6 (1.0)	15 (0.6)	21 (0.7)	0 (0.0)	5 (0.7)	5 (0.5)
**Metastases in mediastinal lymph nodes**						
**No**	268 (87.0)	1489 (86.8)	1757 (86.9)	157 (87.7)	469 (92.1)	626 (91.0)
**Yes**	34 (11.0)	207 (12.1)	241 (11.9)	25 (14.0)	43 (8.4)	68 (10.0)
**Missing**	324 (51.8)	781 (31.6)	1105 (35.6)	116 (38.9)	204 (28.5)	320 (31.6)
**Metastases in cervical/supraclavicular/jugular lymph nodes**						
**No**	327 (93.2)	1645 (89.5)	1972 (90.1)	179 (95.7)	501 (92.8)	680 (93.5)
**Yes**	18 (5.1)	163 (8.9)	181 (8.3)	8 (4.3)	32 (5.9)	40 (5.5)
**Missing**	281 (44.9)	669 (27.0)	950 (30.6)	111 (37.2)	183 (25.5)	294 (29.0)

In Sem patients, the distribution of IGCCCG prognostic groups, sites of metastases and tumour markers were similar in de novo metastatic patients or patients relapsing from CSI. As the vast majority of Sem patients were in the good prognostic group (96.3%) (Table 1), the analyses in Sem patients were not stratified by the IGCCCG risk group. However, 4% of Sem relapsed with intermediate prognosis features.

Information on prior treatment given for CSI disease was available for 89.6% and 94.9% of NSem and Sem patients, respectively. As expected, 537/561 (96%) of NSem and 234/283 (83%) of Sem CSI patients in whom such information was available were followed by active surveillance (Supplementary Table 1). However, 17.3% of Sem patients had received adjuvant treatment, of which radiotherapy was predominant [22/283 (7.7%)]. Of note, only 1.1% of relapsing NSem patients had received adjuvant bleomycin, etoposide and cisplatin (BEP). Treatment for metastatic disease was similar between patients relapsing from CSI and de novo metastatic disease, with more than 99% of patients receiving conventional-dose combination chemotherapy (Supplementary Table 1).

**Fig. 2:**
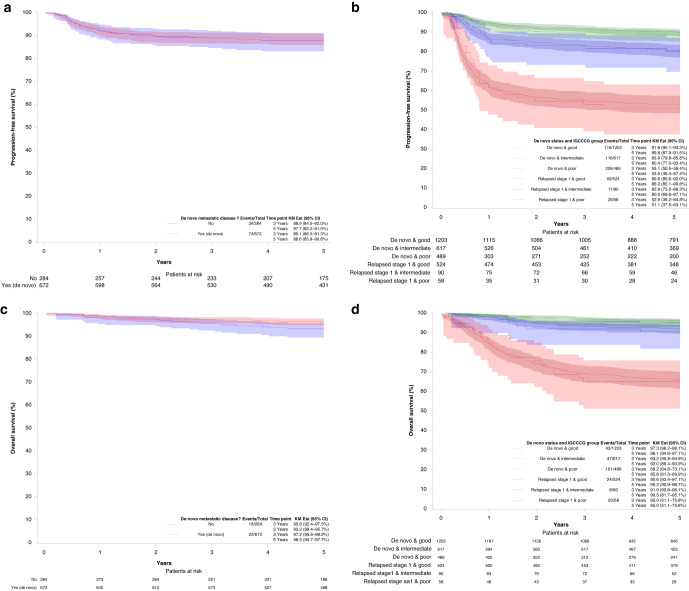
Survival probabilites in seminoma and non-seminoma. Progression-free survival (**a**, **b**) and overall survival (**c**, **d**) of seminoma patients (left) and non-seminoma patients (right). In the seminoma cohort, only patients in the good prognostic group are included. Patients with non-seminoma are divided into good, intermediate and poor prognostic groups.

Among Sem, the overall 5y-PFS and 5y-OS probabilities were similar in patients relapsing from CSI versus de novo metastatic disease in Sem (Fig. 2a, c). The PFS HR of de novo metastatic patients versus patients relapsing for CSI was 0.92 (95% CI: 0.62–1.37, *p*-value = 0.70). In NSem, the PFS and OS were similar within the IGCCCG group (Fig. 2b, d). In NSem without adjustment for the IGCCCG prognostic groups, the 5y-PFS and 5y-OS were significantly higher in relapsed CSI as compared to de novo metastatic patients [84.6% versus 80.0%, HR (de novo/relapsing CSI) = 1.36, 95% CI: 1.09–1.69, *p*-value = 0.007 for PFS, and 93.3% versus 88.7%, HR (de novo/relapsing CSI) = 1.73, 95%CI: 1.24-2.41, *p*-value=0.001, for OS; Supplementary Figures] owing to the higher number of IGCCCG good prognosis patients in the relapsing CSI cohort. Once stratified for IGCCCG prognostic group, the HR for 5y-PFS and 5y-OS in NSem was no longer significant, with a HR of 0.89 (95% CI: 0.70–1.12, *p*-value = 0.30) for PFS and a HR of 0.97 (95% CI: 0.69-1.38, *p*-value = 0.88) for OS, respectively.

## Discussion

The main finding of the present analysis is that PFS and OS are similar in GCT patients relapsing from initial CSI as compared to patients with de novo metastatic disease for Sem patients as well as within the same prognostic IGCCCG subgroups for NSem patients even in a multinational, multi-institutional setting. In the entire cohort, PFS and OS probabilities for NSem were higher for relapsing CSI patients as compared to de novo metastatic patients. However, it is of concern that 18% of patients with metastatic NSem relapsing from CSI had intermediate or poor prognostic features and required more intensive treatment to be cured. This corresponds to 4% of patients with metastatic Sem who fell into the intermediate prognosis category. Only 15% of intermediate and poor relapses occurred later than 3 years after orchiectomy.

An important prerequisite for the success of active surveillance is that patients are fully compliant and are followed with sufficient frequency and vigilance to ensure the detection of relapses early in order to minimise treatment morbidity and disease-related mortality. Two Danish studies documented that a well-structured surveillance strategy applied for CSI patients can ensure early detection of relapses [4, 5]. With controlled follow-up in these Danish studies, 94.4% of NSem patients relapsing after CSI NSem belonged to the good prognostic group, 4.7% to the intermediate prognostic group and only 0.8% to the poor prognostic group. These figures compare to 82.1%, 11.0% and 6.9% of patients in the present study, respectively. Correspondingly, in Sem >99% of patients relapsing from CSI were in the good prognostic group in the Danish study compared to only 96.1% in the present analysis [4, 5].

The present study does not identify the exact reasons for the discrepancy between the present findings and previous reports. One explanation might be the less structured follow-up of some CSI patients in the broader and multinational database that was used for the present analysis. Yu et al. and others have shown that follow-up recommendations developed at referral centres are not always being adhered to in the community [14]. In addition, non-compliance with follow-up recommendations might be frequent among a young and mobile male population [15, 16]. A referral bias with patients being referred to centres contributing to the database from other sites because of intermediate or poor prognosis features or enrolment into high-risk trials may be a confounding factor in the present analysis that we could not eliminate completely. Such a scenario can be a contributing factor to the high number of intermediate/poor prognosis relapses. The lack of international consensus concerning optimal follow-up schedules for CSI patients could also be a contributing factor, which may lead to insufficient follow-up offered to CSI patients in countries with limited access to high-level follow-up care. This can be a problem in particular in tumour marker-negative patients where follow-up relies on clinical and radiological monitoring, which remains insensitive and cumbersome. MicroRNA-371 is a highly sensitive and specific blood-based biomarker that has the potential for earlier diagnosis of relapse but needs further validation [17].

In the present analysis, adjuvant treatment or RPLND was initially administered in 4.3% of CSI NSem patients and in 17.3% of Sem patients in whom this information was available. Moreover, 7.7% of CSI Sem received adjuvant radiation therapy, which is no longer recommended in current treatment guidelines [18, 19]. In retrospective series, a worse prognosis at relapse has been identified in patients relapsing after adjuvant treatment for CSI as compared to those with de novo metastatic disease, which speaks in favour of active surveillance as the preferred management option as was pursued in the majority of patients in the present analysis [20, 21].

Active surveillance is attractive and has become the standard management option in CSI GCT in many countries. This strategy spares further cancer treatment for many patients, but optimal follow-up schedules have yet to be defined. Overly tight schedules may result in lead-time bias, expose patients to unnecessary medical interventions, impair patients’ quality of life and lead to unnecessary costs. However, as shown by the present analysis, unstructured and insufficiently stringent follow-up may result in intermediate or poor prognosis relapses. The present analysis does not resolve this conundrum but may serve as a reminder that active surveillance has to be given careful attention.

In conclusion, the present analysis included a large patient cohort and broad representation from cancer centres worldwide and excluded data obtained from clinical trials. Thus, the results might be close to clinical reality in many countries. It is reassuring that we found no differences in PFS or OS in patients relapsing from initial CSI as compared to de novo metastatic patients with the same IGCCCG prognostic group, demonstrating that active surveillance is safe. However, about 18% of NSem patients and 4% of Sem patients relapsed from initial CSI with intermediate or poor prognosis, which is more than expected from previous reports and exposes those patients to more intensive treatments. Follow-up schedules for active surveillance need to strike the balance of not being unnecessarily tight and not missing out on relapses in time.

### Supplementary information

Supplementary Tables

Supplementary Figures

## Data Availability

Source data will not be made publicly available.
