# Supplementary material for: Outcomes of relapsed clinical stage I versus de novo metastatic testicular cancer patients: an analysis of the IGCCCG Update database
Source: Br J Cancer. 2023 Sep 30;129(11):1759–65. doi: 10.1038/s41416-023-02443-3 (PMC10667594; doi:10.1038/s41416-023-02443-3)
Supplement: Supplementary file 1 — Supplementary Tables [file 41416_2023_2443_MOESM1_ESM.docx]

| **Supplementary Table 1: Treatment regimens** | | | | | | | |
| --- | --- | --- | --- | --- | --- | --- | --- |
|  | **Non-Seminoma** | | | **Seminoma** | | |  |
|  | **Relapsed stage 1 (N=626)** | **De novo (N=2477)** | **Total (N=3103)** | **Relapsed stage 1 (N=298)** | **De novo (N=716)** | **Total (N=1014)** |  |
| **Prior treatment for stage 1 disease** |  |  |  |  |  |  |  |
| **active surveillance** | 537 (85.8) |  |  | 234 (78.5) |  |  |  |
| **radiotherapy** | 2 (0.3) |  |  | 22 (7.4) |  |  |  |
| **carboplatin** | 2 (0.3) |  |  | 10 (3.4) |  |  |  |
| **BEP** | 6 (1.0) |  |  | 1 (0.3) |  |  |  |
| **RPLND** | 7 (1.1) |  |  | 1 (0.3) |  |  |  |
| **Other** | 7 (1.1) |  |  | 15 (5.0) |  |  |  |
| **Missing** | 65 (10.4) |  |  | 15 (5.0) |  |  |  |
| **Type of treatment** |  |  |  |  |  |  |  |
| **Conventional chemotherapy** | 624 (99.7) | 2454 (99.1) | 3078 (99.2) | **298 (100.0)** | **716 (100.0)** | **1014 (100.0)** |  |
| *3xBEP* | 308 (49.4) | 936 (38.1) | 1244 (40.4) | 128 (43.0) | 306 (42.7) | 434 (42.8) |  |
| *3xEP or more* | 46 (7.4) | 152 (6.2) | 198 (6.4) | 90 (30.2) | 224 (31.3) | 314 (31.0) |  |
| *4xBEP or more* | 237 (38.0) | 1189 (48.5) | 1426 (46.3) | 74 (24.8) | 181 (25.3) | 255 (25.1) |  |
| *3xVIP or more* | 15 (2.4) | 30 (1.2) | 45 (1.5) | 2 (0.7) | 2 (0.3) | 4 (0.4) |  |
| *3xTBEP or more* | 8 (1.3) | 66 (2.7) | 74 (2.4) | 0 (0.0) | 2 (0.3) | 2 (0.2) |  |
| *3xTIP or more* | 2 (0.3) | 2 (0.1) | 4 (0.1) | *0 (0.0)* | *0 (0.0)* | *0 (0.0)* |  |
| *BEP + VIP and/or TIP (minimum 3 cycles total)* | 1 (0.2) | 3 (0.1) | 4 (0.1) | *0 (0.0)* | *0 (0.0)* | *0 (0.0)* |  |
| *BEP + EP (minimum 3 cycles total)* | 1 (0.2) | 5 (0.2) | 6 (0.2) | 4 (1.3) | 1 (0.1) | 5 (0.5) |  |
| *CBOP/BEP* | 4 (0.6) | 64 (2.6) | 68 (2.2) | *0 (0.0)* | *0 (0.0)* | *0 (0.0)* |  |
| *GETUG13 dose dense* | 1 (0.2) | 7 (0.3) | 8 (0.3) | *0 (0.0)* | *0 (0.0)* | *0 (0.0)* |  |
| *BOP/VI(P)* | 1 (0.2) | 0 (0.0) | 1 (0.0) | *0 (0.0)* | *0 (0.0)* | *0 (0.0)* |  |
| **High dose chemotherapy*** | 2 (0.3) | 23 (0.9) | 25 (0.8) | **0 (0.0)** | **0 (0.0)** | **0 (0.0)** |  |
| *HD-CE* | 2 (100.0) | 8 (34.8) | 10 (40.0) | *0 (0.0)* | *0 (0.0)* | *0 (0.0)* |  |
| *HD-VIP* | 0 (0.0) | 6 (26.1) | 6 (24.0) | *0 (0.0)* | *0 (0.0)* | *0 (0.0)* |  |
| *HD-not further specified* | 0 (0.0) | 9 (39.1) | 9 (36.0) | *0 (0.0)* | *0 (0.0)* | *0 (0.0)* |  |

Abbreviations: BEP, Bleomycin Etoposide Cisplatin; CBOP, Carboplatin Bleomycin Vincristine Cisplatin; CE, Carboplatin Etoposide; EP, Etoposide Cisplatin; HD, High dose; TBEP, Paclitaxel Bleomycin Etoposide Cisplatin; TIP, Paclitaxel Ifosfamide Cisplatin; VIP, Etoposide Ifosfamide Cisplatin;

*Any patient who received at least one cycle of high dose chemotherapy was classified among the high dose chemotherapy category (e.g. a patient receiving 2 x BEP + 1 x HD-VIP was classified as HD-VIP)
