# Supplementary figures and images for: Outcomes of relapsed clinical stage I versus de novo metastatic testicular cancer patients: an analysis of the IGCCCG Update database
Source: Br J Cancer. 2023 Sep 30;129(11):1759–65. doi: 10.1038/s41416-023-02443-3 (PMC10667594; doi:10.1038/s41416-023-02443-3)

**Supplement Figure A**

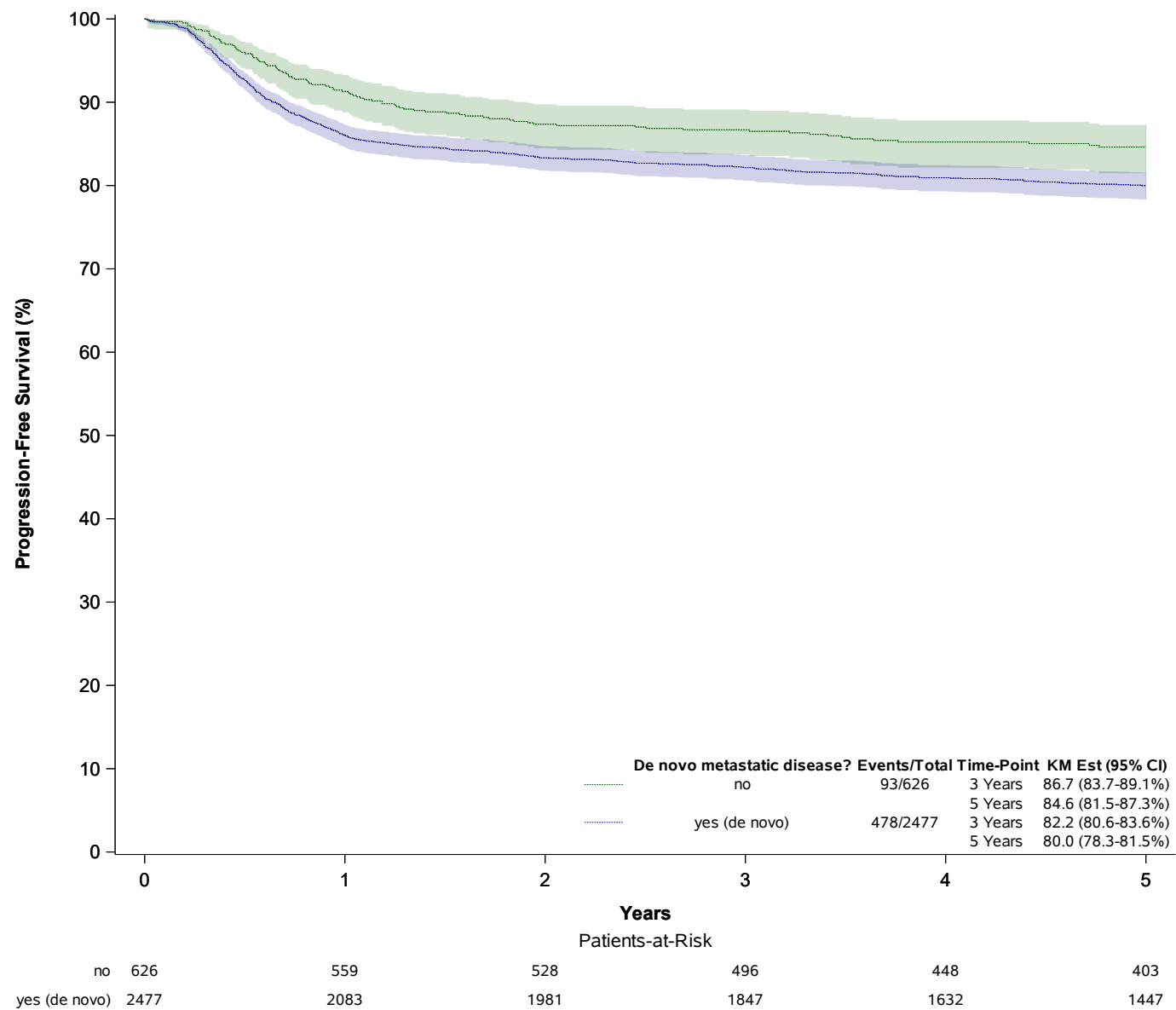

Supplement Figure B

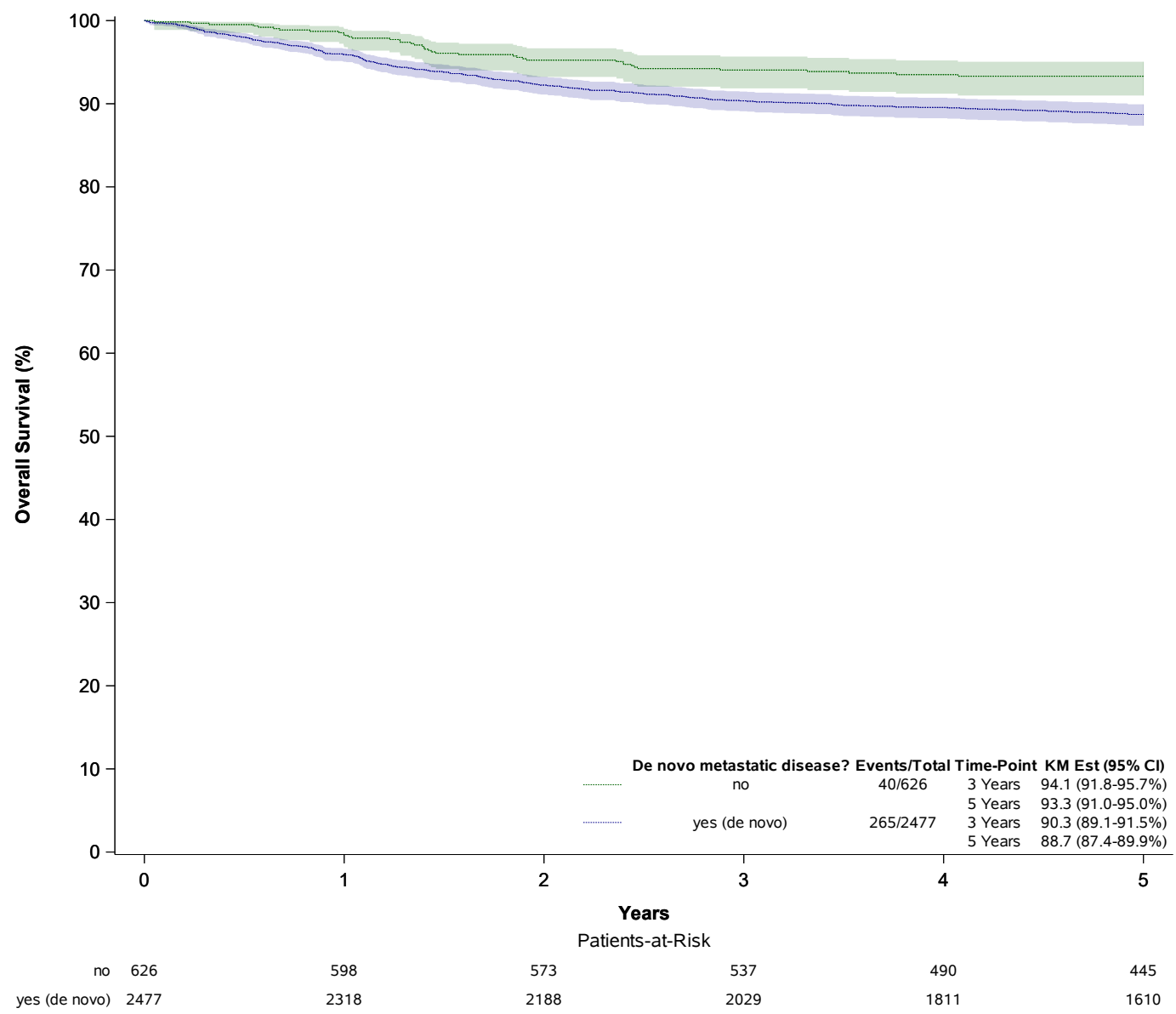

Supplement: Supplementary file 2 — Supplementary Figures [file 41416_2023_2443_MOESM2_ESM.pdf]
